# Nitrous oxide/oxygen plus acetaminophen versus morphine in ST elevation myocardial infarction: open-label, cluster-randomized, non-inferiority study

**DOI:** 10.1186/s13049-020-00731-y

**Published:** 2020-05-12

**Authors:** Sandrine Charpentier, Michel Galinski, Vincent Bounes, Agnès Ricard-Hibon, Carlos El-Khoury, Meyer Elbaz, François-Xavier Ageron, Stéphane Manzo-Silberman, Louis Soulat, Frédéric Lapostolle, Alexandre Gérard, Delphine Bregeaud, Vanina Bongard, Eric Bonnefoy-Cudraz, for the SCADOL II investigators, Vincent Bounes, Claire Vallenet, Elise Robeley, Frédéric Lapostolle, Catherine Pradeau, Patrice Serre, Carols El Khoury, Pascal Usseglio, Eric Revue, Christine Bregeaud, Christine Lespiaucq, Sonja Curac, Julie Jardon, Pierre Arnaud Fort, Armelle Severin, Guillaume Debaty, Anne-Sophie Lucas, Bahram Chaybany, Alexandre Gerard, Marc Fournier, Anais Bauer, Mustapha Sebbane, Tahar Chouihed, Camille Machet, Julie Labiau, Claire Broche, Céline Maisondieu, Matthieu Marchetti, Agnès Ricard-Hibon, François-Xavier Ageron, Nicolas Bohrer, Laurent Teillol, Muriel Vergne, Didier Dansou, Dominique Cailloce, David Sapir

**Affiliations:** 1 Emergency Department, Toulouse University Hospital, INSERM UMR 1027, University Toulouse III Paul Sabatier, Toulouse, France; 2 Emergency Department, Rangueil University Hospital, 1 Av. Jean Poulhès, 31059 Toulouse, France; 3 Emergency Department - SAMU 33, CHU de Bordeaux; INSERM U1219 - Injury Epidemiology Transport Occupation” team, University Bordeaux II, 33000 Bordeaux, France; 4 SAMU31, Toulouse University Hospital; University Toulouse III Paul Sabatier, Toulouse, France; 5 Pôle Emergency Department, SAMU − Centre Hospitalier René Dubos Pontoise, 95300 Pontoise, France; 6 Emergency Department and RESCUe Network, Lucien Hussel Hospital, Vienne, France; 7 Univ. Lyon, Claude Bernard Lyon 1 University, HESPER EA, 7425 Lyon, France; 8 Department of Cardiology, Rangueil University Hospital, Toulouse, France; 9 Emergency Department, Centre Hospitalier Annecy Genevois, Annecy, France; 10 Cardiology department, Lariboisire Hospital, APHP, Paris, France; 11 Paris VII University UMRS 942, Paris, France; 12 SAMU 35 SMUR Urgences adultes, Centre Hospitalier Universitaire Rennes, Université Rennes 1, Rennes, France; 13 SAMU 93 - UF Recherche-Enseignement-Qualité Université Paris 13, Sorbonne Paris Cité, Inserm U942 Hôpital Avicenne, AP-HP, 125, rue de Stalingrad, 93009 Bobigny, France; 14 Hospices Civils de Lyon SAMU 69 - Hôpital Édouard HERRIOT 5, place d’Arsonval, 69437 LYON Cedex 03, France; 15 Hospital Centre of Chateauroux, Chateauroux, France; 16 Department of Epidemiology, Centre Hospitalier Universitaire de Toulouse, Toulouse, France; 17 Department of Public Health, Université Toulouse 3; UMR 1027 INSERM - Université Toulouse 3, Toulouse, France; 18 Hôpital cardiologique Louis-Pradel, 69500 Lyon, France; 19 Université Lyon-1, 69100 Lyon, France

**Keywords:** ST-segment elevation myocardial infarction, Analgesia, prehospital

## Abstract

**Background:**

Studies have shown disparate results on the consequences of morphine use in ST-segment elevation myocardial infarction (STEMI). No study has evaluated alternative treatments that could be at least non-inferior to morphine without its potentially damaging consequences for myocardial function and platelet reactivity. The aim of this study was to evaluate whether nitrous oxide/oxygen plus intravenous acetaminophen (NOO-A) is non-inferior to morphine to control chest pain in STEMI patients.

**Methods:**

This multicenter, open-label, cluster-randomized, controlled, non-inferiority study compared NOO-A with morphine in 684 prehospital patients with ongoing suspected STEMI of < 12 h duration and a pain rating score ≥ 4. The primary endpoint was the proportion of patients achieving pain relief (numeric rating score ≤ 3) after 30 min. Secondary safety endpoints included serious adverse events and death at 30 days.

**Results:**

The median baseline pain score was 7.0 in both groups. The primary endpoint occurred in 51.7% of the NOO-A group and 73.6% of the morphine group (absolute risk difference − 21.7%; 95% confidence interval − 29.6 to − 13.8). At 30 days, the rate of serious adverse events was 16.0 and 18.8% in the NOO-A and morphine groups respectively (p = NS). The rate of death was 1.8% (NOO-A group) and 3.8% (morphine group) (p = NS).

**Conclusion:**

Analgesia provided by NOO-A was inferior to morphine at 30 min in patients with acute STEMI in the prehospital setting. Rates of serious adverse events did not differ between groups.

**Trial registration:**

ClinicalTrials.gov: NCT02198378.

## Background

Pain can be particularly intense in ST-segment elevation myocardial infarction (STEMI), leading to tachycardia, increased stress, higher workload of the heart and damaging effects on the myocardium [1]. Analgesia, administered as soon as possible after symptom onset, is therefore of paramount importance. Opioids (most commonly morphine) are recommended, [2] although their efficacy and safety have not been fully evaluated in randomized trials. Recently, the deleterious effect of morphine on inhibition of platelet reactivity in STEMI patients treated with P2Y_12_ inhibitors has been reported [3–6]. Studies have reported that morphine is associated with a delayed onset of action of oral antiplatelet drugs due to vomiting or delayed gastric emptying, which reduce the absorption of these drugs [4].

Nitrous oxide/oxygen gas as an equimolar mixture is widely used in emergency medicine and has been tested in acute myocardial infarction [7]. It acts by activating opioid neurons, leading to activation of the descending noradrenergic inhibitory pathways that inhibit nociception [8]. It has minor, rapidly reversible secondary effects and no reported haemodynamic effects [9]. Few studies in emergency medicine have compared Nitrous Oxid-Oxygen to morphine with heterogenous results [10–12]. Acetaminophen is an effective and safe painkiller for emergency department patients [13]. It has been successfully used in multimodal analgesia especially postoperative analgesia [14]. Nitrous oxide/oxygen plus intravenous acetaminophen (NOO-A) could therefore be a suitable alternative to morphine. The association nitrous oxid-oxygen plus intravenous acetaminophen was chosen for reasons of delay in action. NOO has a 3–5 min onset of action and intravenous acetaminophen reaches its peak concentration at the end of the 15-min infusion. Thereafter, the duration of action of acetaminophen is 6 h while the effect of nitrous oxid-oxygen stops 5 min after inhalation is stopped. Thus, Acetaminofen allowed continuing the pain management once NOO was stopped.

The aim of the SCADOL II study was to assess in patients with acute STEMI managed in the prehospital setting the non-inferiority in achieving analgesia at 30 min of an equimolar mixture of NOO plus intravenous acetaminophen compared with intravenous morphine. The secondary safety objectives were the rates of serious adverse events and death at 30 days.

## Methods

### Study design

SCADOL II was a multicenter, open-label, cluster-randomized, controlled, non-inferiority trial. Thirty-eight mobile intensive care unit centers were randomized (1:1) to perform analgesia with NOO-A or intravenous morphine. A computerized randomization process was used to generate the random allocation sequence and was carried out by the methodologist from the list provided by participating centers. The details of the SCADOL II investigators list is provided in Additional file 1: Appendix 1.

The study has complied with the Declaration of Helsinki, the locally appointed ethics committee has approved the research protocol and informed consent has been obtained from the subjects (or their legally authorized representative).

### Selection of participants

Patients aged ≥18 years with suspected STEMI managed by an emergency physician in a mobile intensive care unit were eligible if they had symptom duration of < 12 h and a pain intensity score, assessed on a numeric rating scale, ≥4 (pain scale range 0–10).

Exclusion criteria were severe haemodynamic, respiratory, or neurological failure; heart failure; known allergy or contraindication to morphine or nitrous oxide; morphine or nitrous oxide administration within previous 4 h; incapacity to self-assess pain intensity on a numeric rating scale; legal guardianship; pregnancy; or air ambulance transport.

The reperfusion strategy (thrombolysis or angioplasty) was chosen by the emergency physicians according to guidelines [15]. To prevent delays in the performance of revascularization, centers were randomly allocated before the start of the study, using a cluster design, to the NOO-A or morphine group.

### Study procedures

Analgesics were started by the emergency physician as soon as possible after enrollment. In the control group, morphine administration was titrated every 5 min according to pain intensity, assessed on a numeric rating scale: a 2 mg bolus (or 1 mg, for body weight < 60 kg) was given for a numeric rating scale score 4–5; and a 3 mg bolus (or 2 mg, for body weight < 60 kg) for a score ≥ 6 [16]. In the intervention group, nitrous oxide/oxygen was administered according to the marketing authorization and under supervision of the emergency physician, and was given for ≥30 min (a minimum of 5 min is necessary to obtain an analgesic effect) [17]. Nitrous oxide/oxygen was combined with 1 g intravenous acetaminophen in the framework of a multimodal analgesia. After 15 min of use, if the pain was still intense (numeric rating scale ≥6) the emergency physician could change the analgesic strategy and use morphine; such patients were considered treatment failures. After 30 min and until arrival at hospital, the emergency physician could change the strategy of analgesia.

### Study endpoints

The primary endpoint was the proportion of patients achieving pain relief with NOO-A (without any morphine administration) or morphine, defined as a pain intensity score on a numeric rating scale ≤3 [12, 18], 30 min after starting analgesia [19].

Secondary endpoints included the rate of pre-specified adverse events in the two groups: respiratory depression, defined as a respiratory rate < 10 cycles per minute, or a respiratory score of ≥1 (see Additional file 1, definitions); nausea; vomiting; sedation (measured by a Sedation Scale score) of ≥2; dizziness; and pruritus.

Data on pain intensity, adverse events, and tolerance (heart rate, non-invasive arterial pressure, pulse oximetry) were collected at baseline, every 5 min up to 30 min after the start of analgesia, and at hospital arrival.

A 30-day safety analysis was done on incidence of serious adverse events and death occurring in the 30 days post-treatment.

### Study oversight

The executive and steering committee oversaw the conduct of the trial and the data analysis. The trial was monitored by a clinical research assistant and the data management was done by a data manager independent of the steering committee. Statistical analysis were performed blinded to treatment allocation. Finally, we completed the CONSORT checklist (Additional file 1: Appendix 2).

### Statistical analysis

We estimated that 684 patients and 38 mobile intensive care unit centers (19 clusters) were needed to assess the non-inferiority of NOO-A to achieve pain relief at 30 min, given an 80% expected proportion of pain achievement in the control group, a 10% non-inferiority margin, a 2.5% one-sided alpha error rate, 80% power, and the cluster design. Analysis were performed using SAS version 9.4 (SAS Institute Inc., Cary, NC, USA). A non-inferiority margin was specified in the protocol as an absolute difference of − 10% in proportion. The outcome was deemed to be non-inferior if the lower limit of the 95% two-sided confidence interval (CI) was greater than the non-inferiority margin. The main effect of NOO-A versus morphine on the primary endpoint was assessed using an unadjusted generalized estimating-equations model with an exchangeable covariance matrix to account for the clustering of patients within centers. The intracluster correlation coefficient was estimated by the correlation parameter of the exchangeable covariance matrix and by using linear mixed effect models with a random center effect and the treatment as fixed effect, as the intracluster correlation coefficient calculated ignoring potential treatment effects may be biased [20]. Additional analysis of the primary endpoint included an adjustment for potential risk factors associated with analgesia (baseline pain score, age, sex, and thrombolysis). Random imputations were performed on the basis of the observed values to replace missing pain scores at baseline. Finally, a pre-specified subgroup analysis was done in patients with a confirmed STEMI diagnosis.

The per-protocol population (i.e. patients evaluable for the primary endpoint without major protocol deviations) was used for the primary endpoint analysis, as recommended for a non-inferiority trial [21]. Secondary analysis were done in the intention-to-treat population (i.e. all patients who entered the study, with the exception of patients from prematurely closed centers); pain scores missing at 30 min were imputed by means of the last-observation-carried-forward method. If no pain score was present after baseline, missing values were taken to indicate failure (i.e. a pain score at 30 min of > 3).

In the safety analysis, the incidence of expected adverse events (i.e. sedation, respiratory depression, vomiting, nausea, pruritus, and dizziness), unexpected adverse events, and adverse events leading to treatment discontinuation in the 30 min following initiation of analgesia were computed. The proportions of patients with ≥1 expected adverse event in the 30 first minutes, and of serious adverse events that occurred in the 30 days post-treatment, were compared between groups using a generalized estimating equation model, to account for clustering.

## Results

### Study population

Between November 2014 and December 2016, 38 centers were randomized to the NOO-A or the morphine strategy (19 in each arm). A total of 684 patients were enrolled and composed the intention-to-treat population (340 in the NOO-A group; 344 in the morphine group), all of whom received the study treatment.

**Fig. 1:**
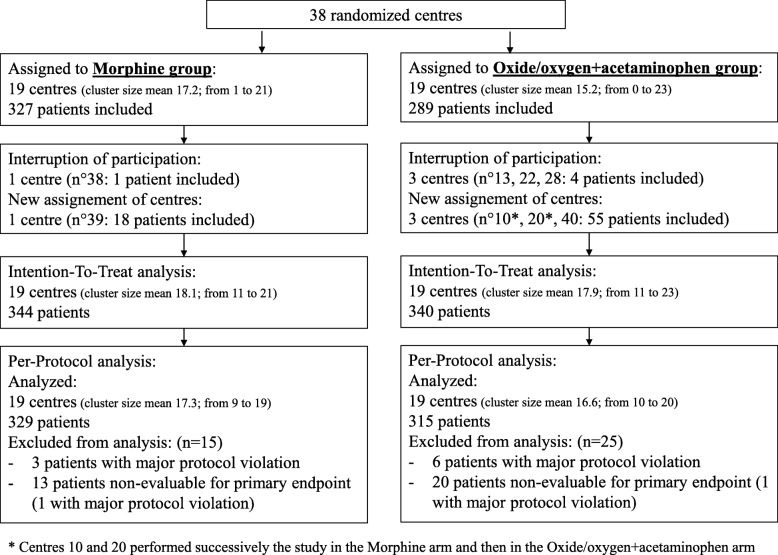
Patient flow chart. Centres 10 and 20 performed the study in the morphine arm and then in the oxide/oxygen plus acetaminophen arm

The per-protocol population comprised 644 patients (315 in the NOO-A group; 329 in the morphine group) (Fig. 1). The patient characteristics were well balanced between groups (Table 1 and Additional file 1: Appendix Table 1). Median pain intensity was 7.0 (interquartile Q1 to Q3: 5.0 to 8.0).

**Table 1:** Characteristics of patients in the per-protocol population

	Nitrous oxide/oxygen plus acetaminophen (*n* = 315)	Morphine (*n* = 329)
Age, years, mean ± SD	61.9 ± 13.7	62.1 ± 13.0
Male sex, *n* (%)	255 (81.0)	249 (75.7)
Body mass index,^a^ kg/m^2^	*n* = 296	*n* = 307
Median (Q1; Q3)	25.8 (23.7; 28.1)	26.0 (23.9; 29.0)
≥30 kg/m^2^, *n* (%)	42 (14.2)	58 (18.9)
Smokers, *n/N* (%)	147/314 (46.8)	150/321 (46.7)
Diabetes,^b^*n/N* (%)	36/312 (11.5)	38/323 (11.8)
Hypertension, ^b^*n/N* (%)	131/312 (42.0)	119/318 (37.4)
Hypercholesterolaemia, ^b^*n/N* (%)	82/305 (26.9)	95/315 (30.2)
Family history of cardiovascular disease, *n/N* (%)	94/274 (34.3)	87/278 (31.3)
Previous coronary artery disease, *n/N* (%)	55/311 (17.7)	56/324 (17.3)
Thrombolysis, *n* (%)	30 (9.5)	46 (14.0)
Decision of angioplasty, *n* (%)	296 (94.0)	308 (93.6)
Treatments at baseline, *n* (%)		
Aspirin	313 (99.4)	323 (98.2)
Other antiplatelet (clopidogrel, ticagrelor or prasugrel)	301 (95.6)	314 (95.4)
Clopidogrel	54 (17.1)	56 (17.0)
Ticagrelor	168 (53.3)	145 (44.1)
Prasugrel	81 (25.7)	116 (35.3)
Heparin	136 (43.2)	163 (49.5)
Low-molecular-weight heparin	159 (50.5)	124 (37.7)
Bivalirudin	21 (6.7)	32 (9.7)
Anticoagulant (heparin, low-molecular-weight heparin or bivalirudin)	307 (97.5)	318 (96.7)
Beta-blocker	4 (1.3)	0
Glycoprotein IIb/IIIa inhibitor	6 (1.9)	9 (2.7)
Anxiolytic	2 (0.6)	10 (3.0)
Other treatment (administered in mobile intensive care unit)	47 (14.9)	69 (21.0)
Delay between chest pain and study treatment start, minutes	*n* = 314	*n* = 329
Median (Q1; Q3)	91.0 (65.0; 161.0)	100.0 (62.0; 167.0)
Pain score on numeric rating scale at study treatment start	*n* = 314	*n* = 328
Median (Q1; Q3)	7.0 (5.0; 8.0)	7.0 (5.0; 8.0)

*Q* quartile, *SD* Standard deviation

^a^Body mass index is the weight in kilograms divided by the square of the height in meters

^b^Treated

### Efficacy

**Fig. 2:**
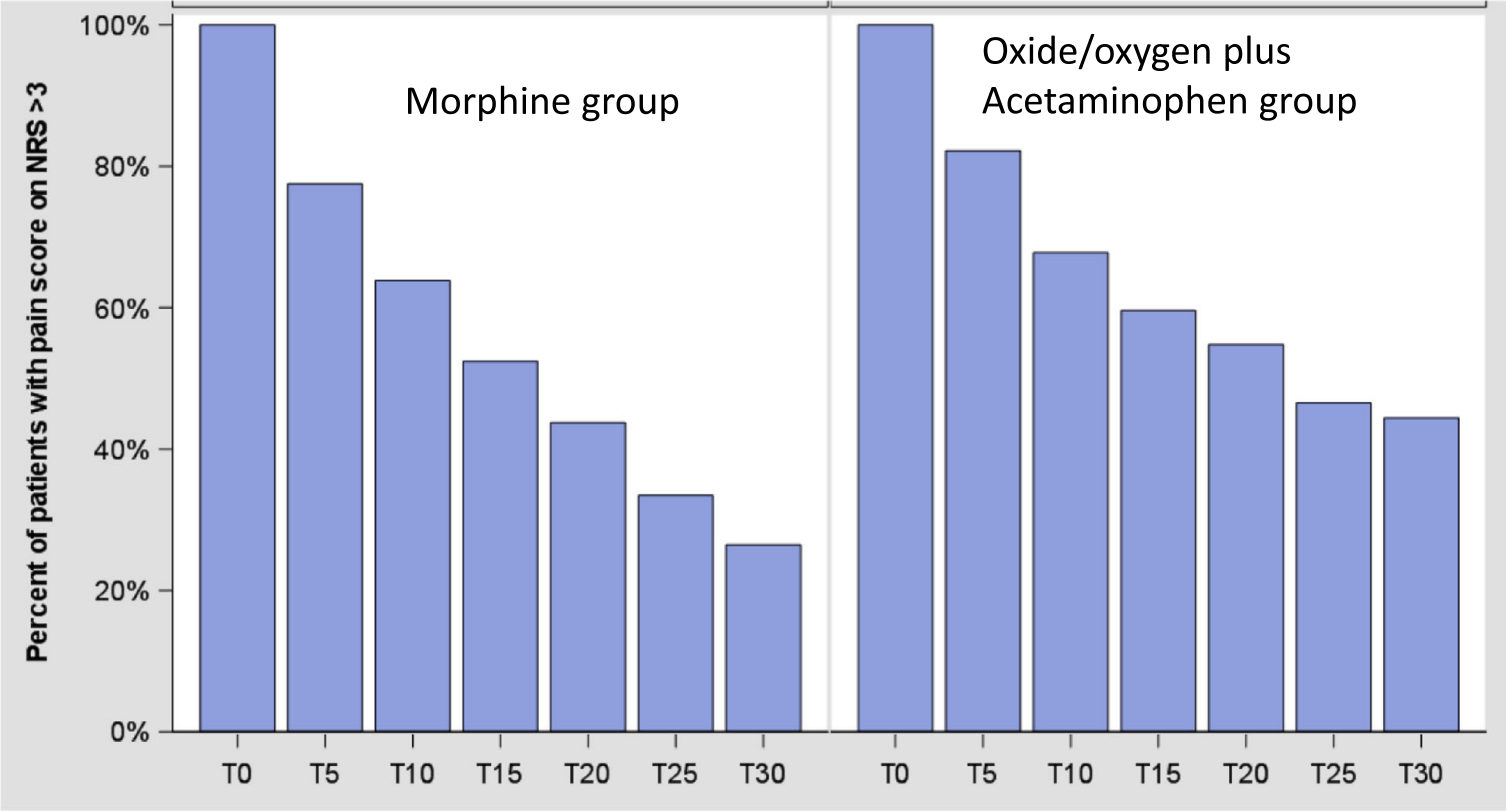
Percent of patients with pain score (numeric rating scale [NRS]) > 3. T, time in minutes

**Fig. 3:**
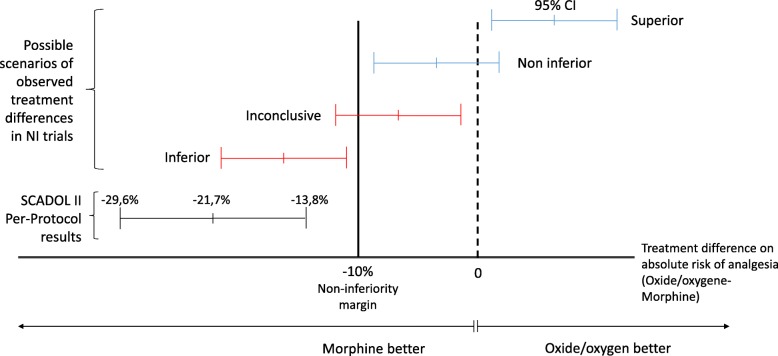
Primary endpoint. CI, confidence interval

Patients in the morphine group were more likely to achieve pain relief than those in the NOO-A group (Fig. 2): the primary endpoint was obtained respectively in 73.6% in the morphine group versus 51.7% of patients in the NOO-A group. The absolute risk difference was − 21.7% [95% Confidence Interval (CI) − 29.6 to − 13.8; *intracluster correlation coefficient* 0.009975), which was below the non-inferiority margin of − 10% defined in the protocol (Fig. 3). Analysis of the primary endpoint in the intention-to-treat population showed the same effect (absolute risk difference − 0.217; 95% CI − 0.297 to − 0.136; intracluster correlation coefficient 0.01518).

A similar observation (more likely to achieve pain relief with morphine) was made in the subgroup of patients with a confirmed diagnosis of STEMI (NOO-A: 144 [50.3%] vs. morphine: 218 [71.7%]), with an absolute risk difference of − 0.21 [95% CI − 0.29 to − 0.13).

Analysis of the primary endpoint in the per-protocol population, adjusted for potential risk factors, showed that better relief of chest pain was associated with morphine treatment, lower pain score at baseline and increasing age (Table 2).

**Table 2:** NOO-A plus acetaminophen versus morphine: adjusted effect on primary endpoint estimated with estimating equations model

Parameter	OR (95% CI)	*P*-Value
Nitrous oxide/oxygen plus acetaminophen (vs. morphine)	0.32 (0.21–0.49)	< 0.0001
Pain score on numeric rating score at baseline (per 1-point increase)	0.58 (0.51–0.67)	< 0.0001
Age (per 1-year increase)	1.02 (1.00–1.03)	0.017
Thrombolysis (vs. none)	0.96 (0.51–1.79)	0.89
Male sex	0.83 (0.58–1.18)	0.29

*NOO-A* Nitrous oxide oxygen, *CI* Confidence interval *OR* Odds ratio

### Safety

The percentage of patients with a predefined adverse event occurring within 30 min of starting analgesia was similar in the two study groups (13.2% with NOO-A and 10.2% with morphine).

(Table 3**)** (absolute risk difference of 0.032 [95% CI − 0.01 to 0.07]; *P* = 0.14). The most frequent expected adverse event was vomiting (5.0% with NOO-A and 4.7% with morphine).

**Table 3:** Incidence of adverse events (intention-to-treat population)

Event, *n* (%)	Nitrous oxide/oxygen plus acetaminophen (*n* = 340)	Morphine (*n* = 344)
Adverse events in the 30 min after starting treatment		
≥1 expected adverse event	45 (13.2)	35 (10.2)
Respiratory depression (< 10 cycles/min or score ≥ R1)	4 (1.2)	5 (1.5)
Nausea (without vomiting)	11 (3.2)	8 (2.3)
Vomiting	17 (5.0)	16 (4.7)
Sedation (score of ≥2)	15 (4.4)	7 (2.0)
Dizziness	3 (0.9)	3 (0.9)
Pruritus	0	3 (0.9)
≥1 unexpected serious adverse event	21 (6.2)	12 (3.5)
Adverse event that led to treatment interruption	24 (7.1)	4 (1.2)
Serious adverse event in the 30 days after enrolment	64 (18.8)	55 (16.0)
Adverse event occurring in ≥1% of patients		
Ventricular tachycardia	21 (6.2)	5 (1.5)
Ventricular fibrillation	8 (2.4)	5 (1.5)
Cardiogenic shock	5 (1.5)	7 (2.0)
Heart failure	3 (0.9)	5 (1.5)
Death in the 30 days after enrolment	6 (1.8)	13 (3.8)

The percentage of patients with an unexpected (not predefined) adverse event within 30 min of starting analgesia was 6.2% with NOO-A and 3.5% with morphine, the most frequent being ventricular fibrillation (1.2% with NOO-A and 0.9% with morphine). The rate of adverse events that led to treatment interruption within the first 30 min was 7.1% with NOO-A and 1.2% with morphine, the most frequent being vomiting (2.9% with NOO-A and 0.3% with morphine).

The incidence of serious adverse event in the 30 days following inclusion was 18.8% with NOO-A and 16.0% with morphine (Table 3) (absolute risk difference of 0.033 [95% CI − 0.030 to 0.096]; *P* = 0.3). The most frequent were ventricular tachycardia (3.8%), ventricular fibrillation (1.9%), cardiogenic shock (1.8%), and heart failure (1.1%). Most cases of ventricular tachycardia (21 out of 26) were observed in the NOO-A group.

Nineteen patients died during the 30 days following inclusion (Additional file 1: Appendix Table 2), 6 in the NOO-A group (1.8%) and 13 in the morphine group (3.8%). None of the deaths were judged to be directly related to the study treatment.

## Limitations

The study has several limitations. Firstly, the low rate of events in both groups made the study underpowered to detect a clinically relevant difference in safety endpoints. It is a common drawback encountered in STEMI studies with prehospital recruitment that select lower risk patients. It does not by itself alter the conclusions of our study. Secondly, even if the cluster randomization provided a pragmatic comparison of chest pain control strategies in the prehospital setting, randomization takes place before consent to participate and individual recruitment. We tried to limit selection bias by a strict monitoring of the study and the rigorous recording of selection criteria. Of note, cluster randomization allowed for the NOO-A treatment to be better included in MICUs routine care. Influence of selection bias cannot be completely ruled out. Thirdly, participants, physicians and patients, were not blinded to the fact that they were receiving morphine or NOO-A. It is therefore possible that a measure reported by patients such as the numeric rating score be influenced by physicians’ pre-existing convictions. From a practical perspective – a double-blind study would have necessitated the transport and management of two gas cylinders in addition to the standard equipment. The open design simplified prehospital logistics and limited treatment delay.

## Discussion

Contrary to our hypotheses, the main finding of our study are that 1) NOO-A was actually inferior to intravenous morphine for the reduction of pain at 30 min in patients with STEMI; 2) there were no more adverse events in the morphine group. Controlling pain at the acute phase of STEMI is challenging. Morphine has been used for years and the issues of its efficacy and its safety have been regularly raised [22, 23]. It has also been linked to a delayed onset of action of oral antiplatelet drugs [4, 5] Few alternatives to morphine have been studied in STEMI, and analgesics that are appropriate for the emergency setting (e.g. non-steroidal anti-inflammatory drugs) are contraindicated [24–26].. The choice of NOO-A combination was especially relevant for use in STEMI. Nitrous oxide/oxygen has short onset of action. It has few unwanted effects [9]. Acetaminophen is an effective and safe painkiller for patients in the emergency department [13]. It was therefore intriguing that the NOO-A combination provided such a relatively low rate of pain relief with only half of the patients expressing a pain intensity less than 3 at 30 min. In contrast, in the morphine group, the proportion of patients with adequate pain relief was high (73%) and consistent with other studies [27, 28]. Intravenous acetaminophen was administered at the recommended dose [16]. This dose proved at least as potent than morphine in patients with renal colitis [9]. It is possible that the NOO-A dosage was suboptimal in view of the high pain intensity of patients with STEMI. Importantly, both treatments were well tolerated. The rate of nausea or vomiting, events that were specifically followed per protocol, was low and did not differ between morphine and NOO-A groups. Morphine is usually considered as responsible of vomiting hence drug interactions, concerns for use in routine. Since NOO-A is not supposed to increase the rate of nausea and vomiting, it may be inferred that the role of morphine in causing nausea and vomiting in STEMI has been overstated. Other factors, especially parasympathetic effect in STEMI with an inferior location and pain intensity may play a more important role. It is clinically relevant because vomiting and delayed gastric emptying have been incriminated in the delayed onset of action of oral antiplatelet drugs in patients treated with morphine [4].

Overall, the rate of adverse events was not significantly different between the NOO-A and the morphine groups. The number of deaths was numerically higher in the morphine group (3.8% vs 1.8% at 1 month). This statistically non-significant higher death rate in the morphine group should not be an argument to renounce to this potent analgesic in STEMI patients. However, this observation adds to the uncertainties surrounding the safety of morphine in ACS even if recent studies have not shown an increase in mortality in patients treated with morphine [5].

## Conclusion

NOO-A was inferior to morphine analgesia at 30 min in patients with acute STEMI in the prehospital setting. Rates of adverse events were not significantly different between the two treatment groups. Because morphine appears to be such a potent agent of pain control in STEMI, a randomised study specifically addressing its safety is warranted.

## Supplementary information

**Additional file 1:Appendix 1.** SCADOL II Investigators list. **Appendix 2.** CONSORT 2010 checklist of information to include when reporting a randomised trial. **Appendix 3.** Definition of secondary outcomes. **Appendix Table 1.** Characteristics of Patients in the Intention-To-Treat Population. **Appendix Table 2.** Characteristics of Patients Who Died Within 30 Days.

## Data Availability

The datasets used and/or analysed during the current study are available from the corresponding author on reasonable request.

## Abbreviations

STEMI: ST-segment elevation myocardial infarction
NOO: Nitrous Oxide/Oxygen
NOO-A: Nitrous oxide/oxygen plus intravenous Acetaminophen
MICU: Medical intensive care unit
ACS: Acute coronary syndrome
