# Supplementary material for: Nitrous oxide/oxygen plus acetaminophen versus morphine in ST elevation myocardial infarction: open-label, cluster-randomized, non-inferiority study
Source: Scand J Trauma Resusc Emerg Med. 2020 May 12;28:36. doi: 10.1186/s13049-020-00731-y (PMC7218609; doi:10.1186/s13049-020-00731-y)
Supplement: Supplementary file 1 — Additional file 1:Appendix 1. SCADOL II Investigators list. Appendix 2. CONSORT 2010 checklist of information to include when reporting a randomised trial. Appendix 3. Definition of secondary outcomes. Appendix Table 1. Characteristics of Patients in the Intention-To-Treat Population. Appendix Table 2. Characteristics of Patients Who Died Within 30 Days. [file 13049_2020_731_MOESM1_ESM.docx]

**Supplementary materials**

# Appendix 1. SCADOL II Investigators list

Vincent Bounes, SAMU 31, Toulouse University Hospital, University Toulouse III Paul Sabatier, Toulouse, France

Claire Vallenet, SMUR Annemasse, Annemasse-Bonneville Hospital, Annemasse, France

Elise Robeley, SAMU 25, Jean Minjoz University Hospital, Besançon, France

Frédéric Lapostolle, SAMU 93, UF Recherche-Enseignement-Qualité Université Paris 13, Sorbonne Paris Cité, Inserm U942 Hôpital Avicenne, AP-HP, Bobigny, France

Catherine Pradeau, SAMU 33, Pellegrin University Hospital, Bordeaux, France

Patrice Serre, SMUR Bourg en Bresse, Flevriat, Bourg en Bresse

Carols El Khoury, Emergency Department and RESCUe Network, Lucien Hussel Hospital, Vienne, France, Univ. Lyon, Claude Bernard Lyon 1 University, HESPER EA 7425, Lyon, France

Pascal Usseglio, SMUR Chambéry, Chambéry Hospital, Chambéry,France

Eric Revue, SMUR Chartres, Chartres Hospital, Chartres, France

Christine Bregeaud, SMUR Chateauroux, Hospital Centre of Chateauroux, Chateauroux, France

Christine Lespiaucq, SAMU 63, Clermont-Ferrand University Hospital, Clermont-Ferrand, France

Sonja Curac, SMUR Beaujon, Beaujon Hospital, Clichy, France

Julie Jardon, SAMU 09, Val d’Ariège Hospital, Foix, France

Pierre Arnaud Fort, SAMU 47, Agen Hospital,Agen, France

Armelle Severin, SAMU 92, Poincaré University Hospital, Garches, France

Guillaume Debaty, SAMU 38, Grenoble University Hospital, Grenoble France

Anne-Sophie Lucas, SAMU 85, La-Roche-Sur-Yon Hospital, La-Roche-Sur-Yon, France

Bahram Chaybany, SAMU 59, Lilles University Hospital, Lilles, France

Alexandre Gerard, SAMU 69, Edouard Herriot Univseristy Hospital, Lyon, France

Marc Fournier, SAMU 13, La Timone Univesity Hospital, Marseille, France

Anais Bauer, SAMU 57, Metz University Hospital, Metz, France

Mustapha Sebbane, SAMU 34, Montpelier University Hospital, Montpellier, France

Tahar Chouihed, SAMU 54, Nancy University Hospital, Nancy, France

Camille Machet, SAMU 44, Nantes University Hospital, Nantes, France

Julie Labiau, SAMU 06, Nice University Hospital, Nice, France

Claire Broche, SMUR Lariboisière, Lariboisière Univesrity Hospital, Paris, France

Céline Maisondieu, SMUR Pitié- Salpêtrière, Pitié- Salpêtrière University Hospital, Paris, France

Matthieu Marchetti, SAMU 86, Poitiers University Hospital, Poitiers, France

Agnès Ricard-Hibon, Pole Emergency Department – SAMU − René Dubos Hospital, Pontoise, France

François-Xavier Ageron, Emergency Department, Centre Hospitalier Annecy Genevois, Annecy, France

Nicolas Bohrer, SAMU 97, Felix Guyon University Hospital, Saint Denis de la Réunion, France

Laurent Teillol, SMUR Saint Gaudens, Comminges Pyrennees Hospital, Saint Gaudens, France

Muriel Vergne, SAMU 83, Toulon Hospital, Toulon, France

Didier Dansou, SAMU 37, Tours University Hospital, Tours, France

Dominique Cailloce, SAMU 87, Limoges University Hospital, Limoges, France

David Sapir, SMUR Corbeil, Sud Francilien Hospital, Corbeil Essonnes, France

Appendix 2. CONSORT 2010 checklist of information to include when reporting a randomised trial

| Section/Topic | Item No | Checklist item | Reported on page No |
| --- | --- | --- | --- |
| Title and abstract | | | |
|  | 1a | Identification as a randomised trial in the title | 1 |
|  | 1b | Structured summary of trial design, methods, results, and conclusions (for specific guidance see CONSORT for abstracts) | 1 |
| Introduction | | | |
| Background and objectives | 2a | Scientific background and explanation of rationale | 2 |
|  | 2b | Specific objectives or hypotheses | 2 |
| Methods | | | |
| Trial design | 3a | Description of trial design (such as parallel, factorial) including allocation ratio | 2 |
|  | 3b | Important changes to methods after trial commencement (such as eligibility criteria), with reasons | NA |
| Participants | 4a | Eligibility criteria for participants | 3 |
|  | 4b | Settings and locations where the data were collected | 3 |
| Interventions | 5 | The interventions for each group with sufficient details to allow replication, including how and when they were actually administered | 3 |
| Outcomes | 6a | Completely defined pre-specified primary and secondary outcome measures, including how and when they were assessed | 4 |
|  | 6b | Any changes to trial outcomes after the trial commenced, with reasons | NA |
| Sample size | 7a | How sample size was determined | 4 |
|  | 7b | When applicable, explanation of any interim analyses and stopping guidelines | NA |
| Randomisation: |  |  |  |
| Sequence generation | 8a | Method used to generate the random allocation sequence | 2 |
|  | 8b | Type of randomisation; details of any restriction (such as blocking and block size) | 2 |
| Allocation concealment mechanism | 9 | Mechanism used to implement the random allocation sequence (such as sequentially numbered containers), describing any steps taken to conceal the sequence until interventions were assigned | 2 |
| Implementation | 10 | Who generated the random allocation sequence, who enrolled participants, and who assigned participants to interventions | 2 |
| Blinding | 11a | If done, who was blinded after assignment to interventions (for example, participants, care providers, those assessing outcomes) and how | 3 |
|  | 11b | If relevant, description of the similarity of interventions | NA |
| Statistical methods | 12a | Statistical methods used to compare groups for primary and secondary outcomes | 4-5 |
|  | 12b | Methods for additional analyses, such as subgroup analyses and adjusted analyses | 5 |
| Results | | | |
| Participant flow (a diagram is strongly recommended) | 13a | For each group, the numbers of participants who were randomly assigned, received intended treatment, and were analysed for the primary outcome | Fig 1 |
|  | 13b | For each group, losses and exclusions after randomisation, together with reasons | Fig 1 |
| Recruitment | 14a | Dates defining the periods of recruitment and follow-up | 6 |
|  | 14b | Why the trial ended or was stopped | 6 |
| Baseline data | 15 | A table showing baseline demographic and clinical characteristics for each group | Table 1 |
| Numbers analysed | 16 | For each group, number of participants (denominator) included in each analysis and whether the analysis was by original assigned groups | 6-7 |
| Outcomes and estimation | 17a | For each primary and secondary outcome, results for each group, and the estimated effect size and its precision (such as 95% confidence interval) | 6-7 |
|  | 17b | For binary outcomes, presentation of both absolute and relative effect sizes is recommended |  |
| Ancillary analyses | 18 | Results of any other analyses performed, including subgroup analyses and adjusted analyses, distinguishing pre-specified from exploratory |  |
| Harms | 19 | All important harms or unintended effects in each group (for specific guidance see CONSORT for harms) | NA |
| Discussion | | | |
| Limitations | 20 | Trial limitations, addressing sources of potential bias, imprecision, and, if relevant, multiplicity of analyses | 7 |
| Generalisability | 21 | Generalisability (external validity, applicability) of the trial findings | 7 |
| Interpretation | 22 | Interpretation consistent with results, balancing benefits and harms, and considering other relevant evidence | 7 |
| Other information | | |  |
| Registration | 23 | Registration number and name of trial registry | 2 |
| Protocol | 24 | Where the full trial protocol can be accessed, if available | Appendix |
| Funding | 25 | Sources of funding and other support (such as supply of drugs), role of funders | Title page |

# Appendix 3. Definition of secondary outcomes

## Respiratory Score^1^

R0: Regular breathing and respiratory rate of 10 or more

R1: Snoring and respiratory rate of 10 or more

R2: Irregular breathing and respiratory rate of 10 or more

R3: Apnea

## Sedation Scale1

0: Patient is awake

1: Patient is with intermittent sleeping

2: Patient is sleeping, awakened by verbal stimulation

3: Patient is sleeping, awakened by tactile stimulation

4: Patient is not aroused by stimulation

# Reference

1. Fletcher D, Pinaud M, Scherpereel P, Clyti N, Chauvin M. The efficacy of intravenous 0.15

versus 0.25 mg/kg intraoperative morphine for immediate postoperative analgesia after

remifentanil-based anesthesia for major surgery. *Anesth Analg* 2000;**90**:666-71.

**Appendix Table 1. Characteristics of Patients in the Intention-To-Treat Population**

|  | Nitrous Oxide/Oxygen plus Acetaminophen (*n* = 340) | Morphine  (*n* = 344) |
| --- | --- | --- |
| Age, years , mean ± SD | 61.5 ± 13.6 | 61.9 ± 13.0 |
| Male sex, *n* (%) | 275 (80.9) | 261 (75.9) |
| Body mass index,^*^ kg/m^2^ | *n* = 321 | *n* = 321 |
| Median (Q1 ; Q3) | 25.8 (23.5 ; 28.1) | 26.1 (23.9 ; 29.2) |
| ≥30 kg/m^2^, *n* (%) | 46 (14.3) | 65 (20.2) |
| Smokers, *n/N* (%) | 163/339 (48.1) | 158/335 (47.2) |
| Diabetes,^§^ *n/N* (%) | 38/337 (11.3) | 40/337 (11.9) |
| Hypertension, ^§^ *n/N* (%) | 139/337 (41.2) | 125/332 (37.7) |
| Hypercholesterolaemia, ^§^ *n/N* (%) | 88/329 (26.7) | 100/329 (30.4) |
| Family history of cardiovascular disease, *n/N* (%) | 99/295 (33.6) | 90/287 (31.4) |
| Previous coronary artery disease, *n/N* (%) | 58/336 (17.3) | 61/339 (18.0) |
| Thrombolysis, *n* (%) | 33 (9.7) | 49 (14.2) |
| Decision of angioplasty, *n* (%) | 320 (94.1) | 322 (93.6) |
| Treatments at baseline, *n* (%) |  |  |
| Aspirin | 338 (99.4) | 338 (98.3) |
| Clopidogrel | 58 (17.1) | 57 (16.6) |
| Ticagrelor | 175 (51.5) | 152 (44.2) |
| Prasugrel | 92 (27.1) | 120 (34.9) |
| Antiplatelet (clopidogrel, ticagrelor or prasugrel) | 323 (95.0) | 326 (94.8) |
| Heparin | 151 (44.4) | 168 (48.8) |
| Low-molecular-weight heparin | 167 (49.1) | 129 (37.5) |
| Bivalirudin | 24 (7.1) | 34 (9.9) |
| Anticoagulant (heparin, low-molecular-weight heparin or bivalirudin) | 330 (97.1) | 330 (95.9) |
| Beta-blocker | 4 (1.2) | 0 |
| Glycoprotein IIb/IIIa inhibitor | 6 (1.8) | 9 (2.6) |
| Anxiolytic | 2 (0.6) | 10 (2.9) |
| Other treatment (administered in mobile intensive care unit) | 50 (14.7) | 75 (21.8) |
| Delay between chest pain and study treatment start, minutes | *n* = 339 | *n* = 344 |
| Median (Q1 ; Q3) | 90.0 (65.0 ; 155.0) | 100.0 (61.5 ; 167.5) |
| Pain score on numeric rating scale at study treatment start | *n* = 337 | *n* = 341 |
| Median (Q1 ; Q3) | 7.0 (5.0 ; 8.0) | 7.0 (5.0 ; 8.0) |

Abbreviations: Q, quartile; SD, standard deviation.

^*^ Body mass index is the weight in kilograms divided by the square of the height in meters.

^§^ Treated.

**Appendix Table 2. Characteristics of Patients Who Died Within 30 Days**

|  | Nitrous Oxide/Oxygen plus Acetaminophen (*n* = 6) | Morphine  (*n* = 13) |
| --- | --- | --- |
| Age, years, mean ± SD | 72.8 ± 10.3 | 74.8 ± 13.3 |
| Male sex, *n* (%) | 4 (66.7) | 8 (61.5) |
| Centre number, *n* (%) |  |  |
| 1 | 0 | 1 (7.7) |
| 3 | 2 (33.3) | 0 |
| 6 | 2 (33.3) | 0 |
| 10 | 1 (16.7) | 0 |
| 11 | 0 | 1 (7.7) |
| 12 | 0 | 1 (7.7) |
| 14 | 0 | 1 (7.7) |
| 19 | 0 | 1 (7.7) |
| 20 | 1 (16.7) | 0 |
| 23 | 0 | 1 (7.7) |
| 32 | 0 | 1 (7.7) |
| 33 | 0 | 2 (15.4) |
| 34 | 0 | 1 (7.7) |
| 35 | 0 | 2 (15.4) |
| 39 | 0 | 1 (7.7) |
| Delay between inclusion and death, days, median (Q1 ; Q3) | 7.5 (1.0 ; 11.0) | 4.0 (2.0 ; 15.0) |
| ST-segment elevation myocardial infarction, *n* (%) | 5 (83.3) | 12 (92.3) |
| Smokers, *n/N* (%) | 3/6 (50.0) | 4/12 (33.3) |
| Diabetes,^*^ *n/N* (%) | 0 | 6 (46.2) |
| Hypertension,^*^ *n/N* (%) | 3 (50.0) | 9 (69.2) |
| Hypercholesterolaemia,^*^ *n/N* (%) | 2/6 (33.3) | 4/12 (33.3) |
| Family history of cardiovascular disease, *n/N* (%) | 2/5 (40.0) | 1/11 (9.1) |
| History of coronary artery disease, *n/N* (%) | 1 (16.7) | 4 (30.8) |
| Reperfusion strategy |  |  |
| Thrombolysis, *n/N* (%) | 1 (16.7) | 2 (15.4) |
| Angioplasty, *n/N* (%) | 5 (83.3) | 12 (92.3) |
| No reperfusion, *n/N* (%) | 0 | 1 (7.7) |
| Delay between chest pain and study treatment start, minutes, median (Q1 ; Q3) | 118.5 (53.0 ; 140.0) | 120.0 (60.0 ; 170.0) |
| Pain score on numeric rating scale at treatment start, median (Q1 ; Q3) | 6.0 (5.0 ; 9.0) | 7.0 (5.0 ; 8.0) |

Abbreviations: Q, quartile; SD, standard deviation.

^*^ Treated.
